# Convergence of two major pathophysiologic mechanisms in nasal polyposis: immune response to *Staphylococcus aureus* and airway remodeling

**DOI:** 10.1186/1916-0216-42-27

**Published:** 2013-03-28

**Authors:** Rogério Pezato, Leonardo Balsalobre, Milena Lima, Thiago F P Bezerra, Richard L Voegels, Luis Carlos Gregório, Aldo Cassol Stamm, Thibaut van Zele

**Affiliations:** 1 Department of Otolaryngology – Head and Neck Surgery of Federal University of São Paulo, Rua Maestro Antão Fernandes, 173, Jd São Bento, São Paulo, SP02526-060, Brazil; 2 Department of Oto-Rhino-Laryngology, Ghent University Hospital, Ghent University, Ghent, Belgium; 3 Military Police Hospital, São Paulo, Brazil; 4 Department of Otorhinolaryngology and Ophthalmology, University of São Paulo, São Paulo, Brazil

**Keywords:** Airway remodeling, Superantigens, Nasal polyps

## Abstract

This review is addressed two pathophysiologic mechanisms implicated in the pathogenesis of nasal polyposis: the unique remodeling process found in nasal polyp tissue and the immune response of patients with nasal polyposis to *Staphylococcus aureus*.

These two theories converge to the same direction in different aspects, including decreased extracellular matrix production, impaired T regulation and favoring of a Th2 immune response.

In patients with nasal polyposis, an exaggerated immune response to *Staphylococcus aureus* may aggravate the airway remodeling process.

## 

In this article, we will discuss two important mechanisms implicated in the pathophysiology of nasal polyposis that have recently received much research attention, and highlight aspects in which these mechanisms intersect.

This review clearly shows the potential for exacerbation of the nasal polyp remodelling process due to an immune response to *Staphylococcus aureus*.

### Characteristics of the airway remodelling process in chronic rhinosinusitis with nasal polyps (CRSwNP)

Many authors consider the lower and upper airways as a continuum [1] of tissue that shares the same pseudostratified ciliated columnar epithelium lining. Furthermore, the “one airway, one disease” concept is widely known and established the world over. Asthma and nasal polyposis essentially share the same inflammatory characteristics: a predominance of eosinophil infiltration, goblet cell hyperplasia, and a Th2-cell immune response [2,3]. The similarities between the upper and lower airway mucosa and the inflammatory processes that affect them, associated with the fact that polyposis in the lung mucosa is virtually unheard of, make the bronchial mucosa an extraordinary model for the study of nasal polyposis [4].

There are some important differences in the remodeling process between the lower and upper airway: nasal epithelial disruption, basement membrane pseudothickening, and elastase-positive cells, which are less numerous in the nasal mucosa than in lung mucosa [5]. Nasal polyposis also features major edema, albumin-filled pseudocysts, and alpha-2-macroglobulin; in CRSwNP, soft tissue edema and overgrowth of nasal mucosa predominate [6]. Conversely, in chronic rhinosinusitis without nasal polyps (CRSsNP), there is mucosal fibrosis with an increase in collagen.

The importance of the epithelium is demonstrated experimentally when injured epithelium results in impaired repair and an increase in the production and release of TGF-ß [7].

TGF-ß1 is a cytokine produced by most immune cells, fibroblasts and many other cells; it is considered a pleiotropic and multifunctional growth factor with important immunomodulatory and fibrogenic effects.

The function of TGF-ß1, however, does not seem to be one-dimensional. A dual anti-inflammatory and profibrotic role may occur [8].

The role of TGF-ß1 expression in nasal polyposis is still controversial. Some authors advocate that TGF-ß1 is increased in nasal polyposis compared to healthy nasal mucosa or CRSsNP, but these studies used immunohistochemistry to support their findings [9,10], while others have reported opposite findings with use of quantitative methods such as Elisa or PCR in nasal tissue homogenates [2,11].

It is also important to consider the fact that, in nasal polyposis, TGF-ß1 expression patterns are different in the stroma and epithelium, being higher and lower respectively as compared with control nasal mucosa.

TGF-ß1 plays an important role in the balance of fibrinolysis and fibrogenesis and, consequently, on the extracellular matrix (ECM).

In NP tissue, matrix metalloproteinase 7 (MMP-7) and matrix metalloproteinase 9 (MMP-9) levels are increased and tissue inhibitor of metalloproteinases 1 (TIMP-1) levels are decreased as compared with normal nasal mucosa [12].

This imbalance can be partially explained by the lack of TGF-ß1 in NP and its inhibitory effect on MMP-9 activity via TIMP-1 release [13].

For the same reason, in NP, TGF-ß_1_-activated PAI-1 (plasminogen activator inhibitor-1) is decreased and, consequently, plasminogen activator and MMP levels are increased as compared with controls and patients with CRSsNP [14].

The hypothesis that CRSsNP is characterized by fibrosis, high levels of TGF-ß and increased Treg activity, whereas NP is characterized by edema, a lack of extracellular matrix, and low TGF-ß levels and Treg activity [4,6] is the cornerstone of the chronic rhinosinusitis remodelling process, according to recent studies.

### Impact of *Staphylococcus aureus* superantigens on chronic rhinosinusitis with nasal polyps

NP is a severe chronic inflammatory disease involving the pnasal sinuses that is frequently associated with asthma and aspirin sensitivity. NP in combination with aspirin-induced asthma (AIA) represents the most severe form of airway inflammation within the group of patients with NP. The pathophysiology of NP is not fully understood, and is most likely to be multifactorial. Over the years, this disease has frequently been linked to staphylococcal colonization and, particularly, to bacterial products such as enterotoxins from *Staphylococcus aureus*.

Staphylococcal species are the most prevalent bacteria that have been isolated from the nasal mucus of white patients with NP. An increased colonization rate of *S. aureus* has been demonstrated in patients with NP (63.6%), but could not be demonstrated in patients with chronic rhinosinusitis without polyps (27.3%) as compared with control subjects. In patients with NP and comorbid asthma or aspirin sensitivity, colonization rates were further increased, up to 80% [15]. *S. aureus* has also been detected in the submucosal space by peptide nucleic acid-fluorescence in situ hybridization (PNA-FISH) in patients with NP, and especially in subgroups of NP with aspirin sensitivity [16,17].

These are both potential reservoirs for superantigen release in the sinuses. Th2-polarized inflammation with a resultant eosinophilic inflammatory milieu has also been linked to the presence of *S. aureus* biofilms. This may occur both dependently and independently of the superantigen pathway, implying a direct link between microorganism and host [18]. A different form of colonization by *Staphylococcus aureus*, through biofilms, has received attention recently.

A biofilm is a group of adherent bacteria irreversibly anchored to a surface and enclosed in a matrix of exopolysaccharide [19,20].

Fungal elements associated with bacteria within biofilms of CRS patients have been demonstrated, an association that could lead to enhanced virulence of bacterial biofilms [21].

Biofilm-grown bacteria can be very resistant to fluctuations in moisture, pH and temperature. They are also highly resistant to antibiotic therapy, which may explain chronic infections refractory to clinical management [19,20].

Foreman et al. confirmed the hypothesis that biofilms would be present in NP patients as a nidus from which planktonic *S. aureus* and superantigens are released into the pnasal sinuses [18].

*Staphylococcus aureus* biofilms in NP patients have been shown to result in eosinophilic inflammation and significantly higher levels of IL-5 and ECP. Staphylococcal superantigen-specific IgE was associated with a Th2-skewed response and a significantly elevated total IgE, IL-5 and ECP. The presence of *Staphylococcus aureus* biofilms was also associated both with worse symptoms and worse Lund-Mackay scores in patients with NP.

Biofilms have also been detected in control groups composed of patients who underwent septoplasty surgery due to nasal obstruction [22].

The mechanisms of this increased bacterial colonization in NP are unclear, but recent data suggest that a defect in the phagocytic system in NP might contribute to increased *S. aureus* colonization [23].

The pathogenic impact of *S. aureus* in NP has been mainly attributed to virulence factors secreted by *S. aureus*, such as staphylococcal enterotoxins. However, screening for staphylococcal superantigens genes showed no correlation with the presence or severity of NP. This suggests that the specific immune response of the host to *S. aureus* colonization, rather than the panel of enterotoxin genes present, determines the pathophysiology of NP [24,25].

*Staphylococcus aureus* enterotoxins (SEs) elicit a massive inflammatory reaction resulting in a polyclonal activation of T and B-lymphocytes independent of the specific adaptive immune response. Clonal expansion of the corresponding V-β signature region of TCRs might play a major role in the pathogenesis of NP. A significant, specific TCR-Vβ expansion linked to the presence of serum IgE to staphylococcal toxins was observed in NP tissue, suggesting a superantigen reaction [26].

SEB stimulation in cultured nasal polyps increases levels of IFN-γ and IL-4, but not of IL-10. It also upregulates mRNA expression of T-bet and GATA-3, but not that of Foxp3 or RORγt, which indicates that SEB is able to affect T_reg_ activity and cause T_reg_ insufficiency. SEB stimulation also increases levels of various pro-inflammatory factors, including IL-2, IL-6, and IL-8, in cultured nasal polyps; these, in turn, also affect T_reg_ activity [27,28].

A second mechanism by which the local inflammatory response can be upregulated is specific IgE directed against *S. aureus* enterotoxins. IgE antibodies to *S. aureus* enterotoxins were present in 28% of polyp samples, with rates as high as 80% in the subgroup of patients with asthma and aspirin sensitivity, as compared with 15% in control individuals and 6% in patients with CRSsNP, respectively [15]. The presence of specific IgE against *S. aureus* enterotoxins was also coincident with higher levels of interleukin IL-5, eotaxin and ECP. Consistent with the increase in IL-5 and other Th2 cytokines, a significant increase in local IgE, IgA and IgG antibodies can be observed in polyp patients. Nasal polyp homogenates in which *S. aureus* enterotoxin–IgE antibodies were detectable had significantly greater concentrations of IgG, IgG4 and IgE than did those without *S. aureus* enterotoxin-specific IgE, positively correlating with IgE and the number of plasma cells, whereas the IgG2 fraction was significantly lower. These changes were not reflected in the serum of patients, confirming the notion of a local impact of superantigens – via direct action on B cells or indirectly via T-cell derived cytokines – on immunoglobulin synthesis. *S aureus* superantigens can also induce the formation of polyclonal IgE directed against multiple inhalant allergens. These polyclonal IgE antibodies in NP are functional and able to activate mast cells ex vivo upon allergen challenge; apart from typical inhalant allergens, SEB may also serve as an allergen per se or as an indicator for superantigen impact on mucosal inflammation by maintaining a continuous activation of mast cells [29,30].

Patients with NP typically exhibit an upregulation of proinflammatory cysteinyl leukotrienes and a downregulation of prostaglandin E2, which is considered an anti-inflammatory metabolite. NP patients who exhibit an immune response to *S. aureus* have an upregulated production of cysteinyl leukotrienes, leukotriene B4 and lipoxin A4 as compared with tissue from nasal polyp patients who were negative for *S. aureus* enterotoxin-specific IgE [31].

Nevertheless, a direct mechanism by which enterotoxins can modify prostanoid metabolism and related functions has yet to be identified.

The presence of specific IgE to staphylococcal enterotoxins is not only associated with a more severe and persistent inflammatory response, but is also linked to clearly identified clinical phenotypes of NP. Rates of both staphylococcal colonization and specific IgE to *Staphylococcus* are increased in NP patients if co-morbid asthma and aspirin intolerance are present [15].

Recently, the presence of IL-5 and IgE antibodies to *S. aureus* enterotoxin (SE-IgE positivity) in human NP patients was shown to be associated with an increased risk of comorbid asthma, suggesting a decisive role of staphylococcal superantigens in amplifying and aggravating airway disease [32].

### The intersection of two paths

The dual role of TGF-ß_1_ – increasing the extracellular matrix and acting as an anti-inflammatory cytokine – may be directly involved in the development of nasal polyposis. The lack of TGF-ß_1_ in nasal polyps could partially explain the soft tissue features (edema and pseudocyst formation) in the matrix and the severe inflammatory process found.

TGF-ß_1_ (and, probably, other members of the transforming growth factor family as well) inhibits activation and proliferation of helper T cells, cytotoxic T cells, macrophages, and monocytes, and reduces the secretion of pro-inflammatory cytokines [33,34]. It also interferes with antibody production by B cells [35].

TGF-ß is implicated in T-cell differentiation. T_reg_ differentiation is favored by TGF-ß, but interleukins associated with pro-inflammatory cytokines (such as IL-21) favor Th17 differentiation [36].

The remodelling aspect in NP is a general phenomenon which is present in all subtypes of polyps (from European subjects, Chinese subjects, SE-mediated and non-SE-mediated polyps), but can be intensified by immune response to *Staphylococcus aureus* superantigens.

The fibrinolytic environment found in NP is worsened by MMP-2. MMP-9 levels have been found to increase after *Staphylococcus aureus* stimulation [37].

As described above, SEs increase Th activity directly via T-bet and GATA-3 expression, but not T_reg_ activity (via Foxp3), and indirectly foster a pro-inflammatory environment, which, together with TGF-ß, can convert T_regs_ into Th cells. In nasal polyps, where there is a severely inflammatory local environment, there is an imbalance of the Th17/T_reg_ ratio in the blood, with an increase in Th17 and decrease in T_regs_[38].

SEs can per se lead to an increase in polyclonal IgE production that cannot be balanced by a TGF-ß_1_-dependent B-cell inhibitory effect, as it is decreased in NP. This “flood” of polyclonal IgE fosters the acquired immune response via dendritic cell FcεRI and Fcγ receptors and, consequently, a Th2 response [39].

The combination of IgE overexpression and lack of TGF-ß can enhance mast cell degranulation [40], increase cysteinyl leukotriene production and eosinophil attraction and, consequently, exacerbate the Th2-mediated inflammatory process.

The overlap of these two pathophysiologic mechanisms clearly demonstrates that the course of nasal polyposis can be exacerbated through the combined action of innate (impaired remodeling of the nasal mucosa, immune responsiveness) and acquired (environmental exposures, such as *Staphylococcus aureus*) factors (Figure 1).

**Figure 1:**
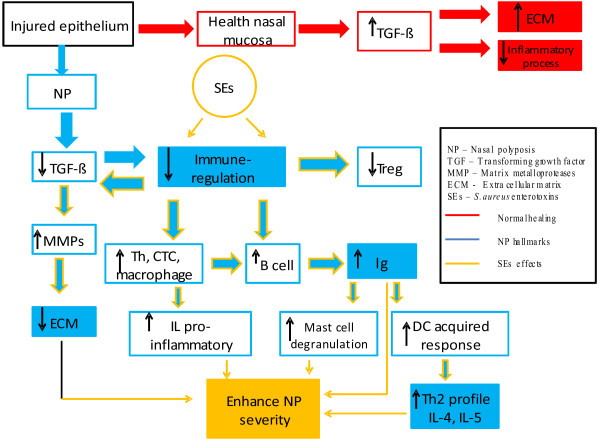
**Illustration of the influence of *Staphylococcus aureus *(in orange) on the remodeling process in nasal polyposis.**

## Competing interests

The authors declare that they have no competing interests.

## Authors’ contribution

RP, TFPB, TvanZ, ML, LB made substantial contributions to study conception and design. RP, TFPB, TvanZ, ML, LB were involved in drafting the manuscript. ACS, RLV, LCG were involved in revising the manuscript critically for important intellectual content. All authors have given final approval for the version to be published.
